# Contrasting Genetic Structure among Populations of Two Amphidromous Fish Species (Sicydiinae) in the Central West Pacific

**DOI:** 10.1371/journal.pone.0075465

**Published:** 2013-10-09

**Authors:** Laura Taillebois, Magalie Castelin, Jennifer R. Ovenden, Céline Bonillo, Philippe Keith

**Affiliations:** 1 Milieux et Peuplements Aquatiques - UMR 7208 (MNHN, CNRS, IRD, UPMC), Muséum national d’Histoire naturelle, Paris, France; 2 Research Institute for the Environment and Livelihoods, Charles Darwin University, Darwin, Northern Territory, Australia; 3 Fisheries and Oceans Canada, Pacific Biological Station, Nanaimo, British Columbia, Canada; 4 Molecular Fisheries Laboratory, University of Queensland, Brisbane, Queensland, Australia; 5 Service de Systématique Moléculaire - UMR 7138, Muséum national d’Histoire naturelle, Paris, France; The Australian National University, Australia

## Abstract

Both present-day and past processes can shape connectivity of populations. Pleistocene vicariant events and dispersal have shaped the present distribution and connectivity patterns of aquatic species in the Indo-Pacific region. In particular, the processes that have shaped distribution of amphidromous goby species still remain unknown. Previous studies show that phylogeographic breaks are observed between populations in the Indian and Pacific Oceans where the shallow Sunda shelf constituted a geographical barrier to dispersal, or that the large spans of open ocean that isolate the Hawaiian or Polynesian Islands are also barriers for amphidromous species even though they have great dispersal capacity. Here we assess past and present genetic structure of populations of two amphidromous fish (gobies of the Sicydiinae) that are widely distributed in the Central West Pacific and which have similar pelagic larval durations. We analysed sections of mitochondrial COI, Cytb and nuclear Rhodospine genes in individuals sampled from different locations across their entire known range. Similar to other Sicydiinae fish, intraspecific mtDNA genetic diversity was high for all species (haplotype diversity between 0.9–0.96). Spatial analyses of genetic variation in *Sicyopus zosterophorum* demonstrated strong isolation across the Torres Strait, which was a geologically intermittent land barrier linking Australia to Papua New Guinea. There was a clear genetic break between the northwestern and the southwestern clusters in *Si. zosterophorum* (φ_ST_ = 0.67502 for COI) and coalescent analyses revealed that the two populations split at 306 Kyr BP (95% HPD 79–625 Kyr BP), which is consistent with a Pleistocene separation caused by the Torres Strait barrier. However, this geographical barrier did not seem to affect *Sm. fehlmanni*. Historical and demographic hypotheses are raised to explain the different patterns of population structure and distribution between these species. Strategies aiming to conserve amphidromous fish should consider the presence of cryptic evolutionary lineages to prevent stock depletion.

## Introduction

Connectivity among populations of amphidromous biota is a consequence of present-day (*i.e.* larval movement during the marine pelagic larval phase) and past (*i.e.* attenuated dispersal due to vicariance) processes. An increasing number of intraspecific phylogeographic studies on marine species have found that earth history events have influenced connectivity between populations [1], [2], [3]. The influence of these events on connectivity is twofold. Oceans are partitioned into biogeographic provinces [4], [5], [6] by physical barriers (*e.g.* Isthmus of Panama) that are a consequence of past geologic activity and which physically limit dispersal. Secondly, eustatic changes in sea level occur over evolutionary time scales, for instance during the Pleistocene period [7], [8], which moderate the effect of geologic barriers and intermittently produced new barriers. Species distributions, and intraspecific genetic structure, directly reflect the influence of these phylogeographic processes over time.

Amphidromy in tropical freshwater fish species is an adaptation for survival in perilous habitats. The freshwater environments of Indo-Pacific islands are generally short, straight and steep [9] with variable flow [10]. The rivers are generally oligotrophic and subject to extreme climatic and hydrological seasonal variations [11]. A distinct, amphidromous, pantropical community of fish (Gobiidae and Eleotriidae) is associated with these habitats [11], [12]. The adults grow, feed and reproduce in streams [11]. After hatching larvae drift downstream into the sea [13], [14] where they spend two to six months [15], [16], [17]. The post-larvae return to rivers, undergo metamorphosis [18], then migrate upstream to settle [11]. By spending part of their life cycle at sea, amphidromous gobies are able to escape insular drought and cyclonic flood events, which cause recurrent local extinctions. The marine larval phase of amphidromous species is generally longer than marine fish [19], [20] and allows dispersal among tropical islands depending on the spatial and temporal characteristics of ocean currents [21]. This leads to high levels of genetic connectivity between populations [9], [22].

In many marine and amphidromous species, major phylogeographic breaks are observed between populations in the Indian and Pacific Oceans, where the shallow Sunda shelf forms a large submerged extension of the continental shelf of mainland Asia. It has constituted an historic geographical barrier to dispersal [23], [24], [25] mainly because it was exposed when sea levels were lower than present [26]. The large spans of open ocean that isolate the Hawaiian or Polynesian Islands are also formidable barriers for most marine [23] species, as well as for amphidromous species even though their pelagic larval durations (PLDs) (and hence dispersal capacity) are high [24], [27], [28]. Such barriers demarcate regions in which connectivity is high. The Central West Pacific bioregion is one such distinct biogeographic province within the Indo-Pacific. It extends from the shallow Sunda shelf to the east of the Malay Peninsula, southwards to the western Islands of Indonesia, then eastwards towards northern Australia and Papua New Guinea. It continues northwards to southern Japan and its eastern extent is bordered by the Melanesian Pacific Islands. It is reputed to have the highest marine biodiversity of the Coral Triangle, and has been the focus of numerous biogeographic studies [29], [30], [31], [32]. Although phylogeographic studies of the Central West Pacific show high connectivity in many reef fish and echinoderms [33], [34], [35], [36], few studies have considered the effect of the Torres Strait as a potential geographic barrier [37].

Little is known about the genetic structure of amphidromous species within the Central West Pacific region [38], especially among species with ranges that span potential biogeographic barriers like the Torres Strait. Here we examine genetic variation within and among *Sicyopus zosterophorum* and *Smilosicyopus fehlmanni*, two widely distributed goby species (Sicydiinae) endemic to the Central West Pacific bioregion. They have similar PLD; about 55 days [15] and biology. Adults of Sicydiinae species show varying habitat preferences such as differing longitudinal distributions along the stream gradients from the lower courses to the upper reaches [39], [40], [41], [42]. However, species from the genera *Smilosicyopus* Watson, 1999 and *Sicyopus* Bleeker, 1857 are mainly found within the middle and upper reaches of rivers and prefer swift, clear and high gradient streams with rocky substrate [43], [44]. Sicydiinae species constitute a large part of fish species biodiversity on tropical islands. These ecosystems are naturally unstable and ephemeral and have become even more so in recent years as result of human alteration [45]. Designing appropriate biodiversity management plans for species that are distributed across large geographic areas on remote islands is dependent on a better understanding of their population structure.

The marine dispersal phase of amphidromous species allows migration between specific habitat patches on remote islands and because dispersal occurs only in the larval stage, this kind of life cycle highlights subtle barriers to dispersal that may not be visible if the adult form was movable from a stream to another. Because of their extended PLD (about 55 days), populations of neither species (*Si. zosterophorum* and *Sm. fehlmanni*) were expected to show genetic breaks within the Central West Pacific bioregion. But, if breaks were present, they would be expected to be congruent because both species have similar PLD, biology and spatial ranges. If the two species present a congruent genetic structure, then it indicates the existence of a phylogeographical barrier within the Central West Pacific bioregion. To test hypotheses of genetic connectivity, we employed mitochondrial and nuclear DNA sequences to assay *Si. zosterophorum* and *Sm. fehlmanni* sampled from seven and five locations, respectively.

## Materials and Methods

### Biological Model

*Sicyopus zosterophorum* (Bleeker, 1857) is a widely distributed species across the Central West Pacific bioregion, covering a longitudinal distance of more than 15,000 km extending from southern Japan and Palau to southern Papua, Vanuatu, New Caledonia and Fiji ([46], [47], [48], [49],personal observations). *Smilosicyopus fehlmanni* (Parenti & Maciolek, 1993) has nearly the same distribution as *Si. zosterophorum* [50] but its precise range has not been determined. *Smilosicyopus chloe* (Watson, Keith & Marquet, 2001) is endemic to New Caledonia and Vanuatu [46], [47]. This latest congeneric was included in the study as a control. First, *Sm. chloe* was used to test whether the two mitochondrial groups of *Si. zosterophorum* were cryptic species or not by comparing the genetic distance within the two congenerics *Sm. chloe* and *Sm. fehlmanni* and the two genetic groups of *Si. zosterophorum*. Also, the two congenerics *Sm. chloe* and *Sm. fehlmanni* co-occur in the rivers of the Vanuatu and New Caledonia islands, showing similar habitat preferences. Their demographic patterns are compared to put forward hypothesis explaining the differences in their ranges. Pelagic larval durations (PLDs) of *Sm. chloe* and *Si. zosterophorum* are 53.6±5.7 and 54.6±5.6 days, respectively [15]. The PLD of *Sm. fehlmanni* is around 54 days [50].

### Sample Collection

**Figure 1:**
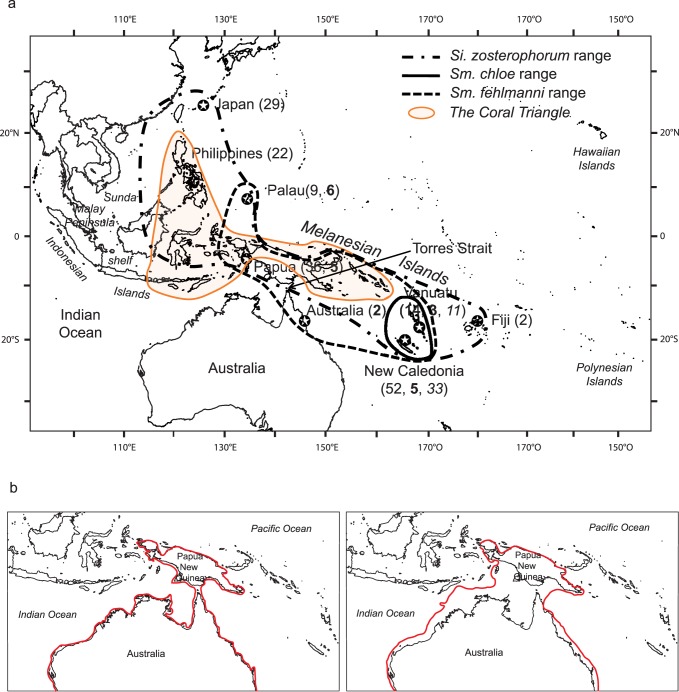
Map of study area (a) and maps depicting sea levels around Australia and Papua region during Pleistocene (b). Collection locations (stars) and sample sizes for *Sicyopus zosterophorum* (sample size in plain text), *Smilosicyopus fehlmanni* (bold text) and *Smilosicyopus chloe* (italics). The 10 m (map on the bottom left) and the 120 m (map on the bottom right) bathymetric contours are indicated by a red line; sea level has fluctuated between these two levels for at least 90% of the time during the Pleistocene (adapted from Mirams *et al*., 2011).

The three Sicydiinae species studied were sampled across their known ranges with repeated visits to many locations and sampling from the upper to lower reaches of rivers. One hundred and sixty five individuals of *Si. zosterophorum* were collected from two regions within the Central West Pacific bioregion: the northwest (Okinawa, Ishigaki and Iriomote Islands, Japan; Philippines; Palau; Papua, Indonesia) and the southwest (New Caledonia; Malekula Island, Vanuatu; Taveuni Island, Fiji). These regions were chosen because they represent the furthest extent of the species’ distribution on either side of a narrow passage that bisects the species ranges. The passage occurs to the south of the Papua New Guinea mainland and northern Australia and is referred to as the Torres Strait. Twenty-one individuals of *Sm. fehlmanni* were caught in the southwest region (Australia; New Caledonia; Pentecost Island, Vanuatu) and the northwest region (Papua, Indonesia; Palau). Forty-four individuals of *Sm. chloe* were collected from the southwest Pacific (New Caledonia; Malekula and Santo Islands, Vanuatu) (Fig. 1a). Geo-referenced collection data is summarized in Table 1.

**Table 1:** Details of sampling of *Sicyopus zosterophorum*, *Smilosicyopus fehlmanni* and *Smilosicyopus chloe*.

Species	Region	Country	River-Island	Latitude	Longitude	Ss	*n*
*Sicyopus zosterophorum*	Southwest Pacific	Vanuatu	Brenwe-Malekula	16°07′35.56″S	167°16′46.77″E	14	14
	–	New Caledonia	Po Vila	20°57′48.22″S	165°18′42.33″E	11	52
	–	–	Newee Dena	20°57′48.70″S	165°19′20.17″E	9	
	–	–	Bas Coulnas	20°40′53.57″S	164°47′33.44″E	2	
	–	–	Wewec	20°36′28.94″S	164°43′57.24″E	26	
	–	–	Pwé Tiéra	20°35′29.92″S	164°44′16.92″E	2	
	–	–	Belep	19°45′37.29″S	163°41′05.25″E	2	
	–	Fiji	Taveuni	16°52′10.55″S	179°54′20.87″O	2	2
	Northwest Pacific	Papua	Kim	04°09′94.30″S	133°09′29.20″E	14	36
	–	–	Bichain	03°42′18.00″S	134°04′41.40″E	8	
	–	–	Kumafa	04°04′14.10″S	133°09′04.40″E	7	
	–	–	Kayumera	03°53′17.40″S	134°28′39.30″E	7	
	–	Palau	Ngmeskang	07°32′48.40″N	134°34′67.00″E	4	9
	–	–	Mesekelat	07°26′30.50″N	134°34′32.10″E	5	
	–	Philippines	Naga-Cebu	10°14′16.62″N	123°43′16.50″E	5	22
	–	–	Salug-Cebu	10°21′16.50″N	123°37′56.85″E	4	
	–	–	Pakil-Laguna	14°29′13.31″N	121°35′03.06″E	13	
	–	Japan	Ishigaki-Jima	24°24′27.85″N	123°09′48.02″E	8	29
	–	–	Okinawa-Jima	26°42′58.36″N	128°16′78.75″E	19	
	–	–	Iriomote-Jima	24°21′57.83″N	123°47′26.28″E	2	
*Smilosicyopus chloe*	Southwest Pacific	Vanuatu	Brenwe-Malekula	16°07′35.56″S	167°16′46.77″E	9	11
	–	–	Santo	15°19′19.06″S	166°53′40.13″E	2	
	–	New Caledonia	Kokengone	20°56′14.33″S	165°17′30.88″E	16	33
	–	–	Newee Dena	20°57′48.70″S	165°19′20.17″E	8	
	–	–	Po Vila	20°57′48.22″S	165°18′42.33″E	2	
	–	–	Wé Djao	20°36′54.40″S	164°44′07.36″E	2	
	–	–	Wewec	20°36′28.94″S	164°43′57.24″E	4	
	–	–	Wan Pwé On	20°31′52.81″S	164°47′02.26″E	1	
*Smilosicyopus fehlmanni*	Southwest Pacific	Vanuatu	Silengwasu-Pentecost	15°39′32.64″S	168°07′20.66″E	3	3
	–	New Caledonia	Kokengone	20°56′14.33″S	165°17′30.88″E	1	5
	–	–	Newee Dena	20°57′48.70″S	165°19′20.17″E	1	
	–	–	Po Vila	20°57′48.22″S	165°18′42.33″E	1	
	–	–	Wé Djao	20°36′54.40″S	164°44′07.36″E	1	
	–	–	Bas Coulnas	20°40′53.57″S	164°47′33.44″E	1	
	–	Australia	Pauls Creek	17°08′55.63″S	145°56′55.65″E	2	2
	Northwest Pacific	Papua	Kim	04°09′94.30″S	133°09′29.20″E	5	5
	–	Palau	Mesekelat	07°26′30.50″N	134°34′32.10″E	1	6
	–	–	Tabecheding	07°27′16.90″N	134°31′74.80″E	2	
	–	–	Tourist wterfall	07°27′48.40″N	134°34′67.00″E	3	

Information includes biogeographic regions, countries, rivers and island, sample sizes at each location (Ss) and size (*n*) of each population.

All specimens were collected by electro-fishing (Portable Dekka 3,000 electric device, Dekka Ltd, Germany) or snorkelling. Either a piece of fin was clipped and the fish was released, or the fish was killed with an overdose of clove oil (10%), and a tissue sample taken. Tissues and fish were stored in 95% ethanol. Specimens and tissue samples were collected under permits of sampling and euthanasia No 60912-2320-2010/JJC, New Caledonia; Marine Research permit (Ministry of Natural Resources) No RE-11-06, Palau; Vanuatu Environment Unit No ENV326/001/1/07/DK and ENV326/001/1/08/DK; LIPI (Indonesian Institute of Science) permit No 43/SI/M26/X/2010; General Fisheries Permit 89212, Environment Protection Agency Permit WITK06337909 and Griffith Animal Ethics Committee approval ENV114/09/AEC and ENV10/09/AEC, Australia.

### DNA Extraction and Amplification

To analyse population structure across the species’ ranges, several gene regions were amplified and sequenced. Total genomic DNA was extracted from pectoral fin tissue with the semi-automated ABI PRISM™ 6100 Nucleic Acid Prep Station following the manufacturer’s instructions. To investigate genetic diversity and patterns of genetic connectivity, a 670 bp fragment of the mtDNA cytochrome oxidase (COI) gene was amplified, for each of the three species, using specific fish primers TelF1-5′TCGACTAATCAYAAAGAYATYGGCAC3′ and TelR1-5′ACTTCTGGGTGNCCAAARAATCARAA3′ [51].

In order to test the population structure observed from the mitochondrial COI data in *Si. zosterophorum*, and to date vicariant events, a 840 bp fragment of mtDNA cytochrome b (Cytb) gene was amplified using specific fish primers CytbF216-5′TCCGAAAYATACATGCYAATGG3′ and CytbR15537-5′CGTTCTGRGCTGAGCTAC3′ [52]. To test species identities (see below), a subset of individuals were randomly selected and sequenced for a fragment of 800 bp of the nuclear rhodopsin (Rh) gene, using the primers F193-5′CARTGGTGCTACCTSTGCGA3′ and R1039-5′CGTGGTCYTTCCKGAAGCG3′ [52]. Likewise, few individuals of *Sm. fehlmanni* and *Sm. chloe* were selected and sequenced as well for this region.

Polymerase chain reactions (PCR) were performed in 25 µL final volume, containing 2.5 µl of the corresponding buffer, 5% of DMSO, 5 µg of bovine serum albumin, 300 µM of each dNTP, 1.7 pM of each of the two primers, 0.3 µM of Taq Polymerase (Qbiogen) and approximately 3 ng of template DNA. Amplification products were generated by an initial denaturation step of 2 min at 94°C followed by 50 cycles of denaturation at 94°C for 20 s, annealing at 52°C for COI, Cytb and Rh for 30 s and extension at 72°C for 60 s with a terminal elongation at 72°C for 3 min. PCR products were purified using Exonuclease I and Phosphatase and sequenced using BigDye Terminator v3.1 kit (Applied Biosystems) and the ABI 3730XL sequencer at Genoscope (http://www.genoscope.cns.fr/) using the same primers. All gene fragments were sequenced in both directions. Chromatograms were edited manually using Sequencher v4.8 (Gene Codes Corporation).

### Species Identity

The species identity of all samples was validated from gene sequences before analysing the patterns of genetic diversity within species. A critical assessment of species identity based on general morphology was also performed using type specimens and specimens of the collection held by the Muséum national d’Histoire naturelle (MNHN) of Paris (France) and the California Academy of Sciences (CAS).

### Phylogeographic Analyses

#### Intraspecific genetic diversity and patterns

Once the species identities of samples were validated, genetic diversity of the COI gene was examined within *Si. zosterophorum* and *Sm. fehlmanni*, and for each population represented by more than five individuals. First, genetic diversity indices were estimated by computing the number of haplotypes [53], number of segregating sites (S, [54]), mean number of pairwise differences (π, [55]) and haplotype diversity (H_d_, [55]) using the software DNAsp v5.10 [56].

Intraspecific genealogical relationships were explored to test the null hypothesis of genetic homogeneity among populations within *Si. zosterophorum* and *Sm. fehlmanni*. The relationships between haplotypes, for both COI and Cytb genes, and their geographic distribution were visualised from a median-joining network (MJN) built using the software Network 4.1.1.2 [57] with equal weights for variable sites.

Hierarchical analyses of molecular variance (amova, [58]) were performed to study the partitioning of genetic variance within and among populations using Arlequin v3.5 [59]. To investigate broader-scale patterns of structuring, a hierarchical AMOVA was performed between regions (*i.e.* northwest and southwest). We also assessed genetic differentiation between each pair of populations by calculating pairwise φ_ST_ values. Significance of F-statistics was calculated from 10,000 replicate analyses based on samples drawn randomly with an alpha value of 0.05.

There was strong evidence of two intraspecific clades (see results for further details) for *Si. Zosterophorum.* To test whether these clades were separate species, the divergence between them was compared to the closely related species *Sm. fehlmanni* and *Sm. chloe*. A neighbour-joining phylogenetic tree was constructed using Kimura two-parameter distances (K2P) [60], [61] from nuclear Rh gene using the software package MEGA v3.1 [62]. The bootstrap analysis was based on 5000 replicates. Two specimens of *Lentipes concolor* (Gobiidae) were used as an outgroup.

#### Demography

We used two analytical techniques to test hypotheses on divergence dates of populations and ages of effective biogeographical barriers among populations of *Si. zosterophorum* and *Sm. fehlmanni*. First, we used mismatch distributions implemented in Arlequin [63], [64]. Mismatch distributions provided τ, the final variable in the coalescence formula and θ_0_ and θ_1_, which are values that, respectively, represent effective female population size at the time of the last common ancestor and the current effective female population size. These parameters were implemented in DNAsp to estimate the frequencies of the nucleotide differences expected under exponential growth and constant population size. The proportion of raggedness values that were as small or smaller than observed values for a given growth rate can be considered as the significance level by which the hypothesis of that level of growth is rejected [64]. Second, Fu’s *F_S_* [65] statistical test of neutrality was performed to test for an excess of recent mutations that are indicative of non-neutral processes such as positive selection or population growth. For each species, Fu’s *Fs* statistics were performed on genetically homogeneous populations. We performed Fu’s *Fs* statistics on the two genetically homogeneous clusters (*i.e.*, northwest clade and the southwest clade) of *Si. zosterophorum* and on *Sm. fehlmanni*. Significantly negative Fu’s *Fs* values are indicative of an expanding population.

#### Biogeography

We used gene divergence time to test hypotheses of vicariance, which predicts that the divergence time between taxa on either side of a barrier should coincide or precede with the timing of the origin of that barrier [66]. The coalescence time of a gene in a group of individuals reflects the age of the most recent common ancestor of that group. A divergence rate of 2% per Myr for Cytb has been widely applied to phylogeographic studies of reef fish and has been found to apply to a goby genus (*Gnatholepis*) (1.95–2.17%, [67]). This genus is a member of the Gobioidei subfamily Gobionellinae and is a sister group to Sicydiinae gobies [68]. *Gnatholepis* is an inhabitant of tropical environments worldwide [69]. We therefore set the divergence rate under a normal prior spanning this range (mean 2.05%, standard deviation 6.122E-4) to obtain a time-scaled phylogeny. We estimated the time to coalescence for standing genetic variation in *Si*. *zosterophorum* samples using the Bayesian Markov Chain Monte Carlo (MCMC) approach as implemented in BEAST 1.7.0 [70]. Models of evolution were computed in jModeltest 0.1 [71], and the GTR+I+Γ model as default priors of mutation processes with unlinked partitions for each codon position. We conducted our analysis with a relaxed uncorrelated lognormal clock and a coalescent model of constant population size [72]. Simulations ran for 50 million generations with sampling every 4000 generations. Three independent runs were computed and runs were then combined with a 10% burn-in using LogCombiner 1.5.2 [70]. Adequate parameter estimation and convergence of chains in the MCMC run were computed in the program TRACER 1.5 [73].

## Results

### Gene Diversity

Of the 164 samples of *Si. zosterophorum* studied, all were sequenced for the COI gene (Table 1), 113 for the Cytb gene and 19 for the Rh gene (Genbank accession number KC407365 - KC407528; KC407252 - KC407364 and KC407233 - KC407251). Twenty-one samples of *Sm. fehlmanni* (Genbank accession number KC407573 - KC407593) and 44 samples of *Si. chloe* (Genbank accession number KC407529 - KC407572) were sequenced for the COI gene. Three samples of *Sm. fehlmanni*, two samples of *Si. chloe* and two samples of *Lentipes concolor* (Genbank accession number KF016034 - KF016040) were sequenced for the Rh gene. After sequence alignment, contigs of 670, 840 and 760 bp in length were obtained for each species for regions of the COI, Cytb and Rh genes, respectively. For each of the three coding genes, no evidence for pseudo-gene amplification was found (no ambiguous alignment, low sequence quality, double peaks or stop codons). All informative substitutions were synonymous in the Rh gene. There were no instances of misidentification of samples used in this study.

For *Si. zosterophorum*, overall haplotype (H_d_) and nucleotide diversity (π) was high (H_d_ *= *0.937; π = 0.0129). H_d_ and π were similar for the northwest (H_d_ = 0.865; π = 0.0098) and for the southwest (H_d_ *= *0.885; π = 0.0039) regions (Table 2). For *Sm. fehlmanni*, overall haplotype (H_d_) and nucleotide diversity (π) was high (H_d_ *= *0.900; π = 0.0036). However, haplotype diversity for Papua population (H_d_ *= *0.7) was lower than for the other populations (Palau H_d_ *= *0.933, New Caledonia H_d_ *= *1) (Table 2). For *Sm. chloe*, overall haplotype (H_d_) and nucleotide diversity (π) was also high (H_d_ *= *0.96; π = 0.046) (Table 2).

**Table 2:** Molecular diversity indices for COI sequences from populations of *Sicyopus zosterophorum*, *Smilosicyopus fehlmanni* and *Smilosicyopus chloe*.

		*Sicyopus zosterophorum*					*Smilosicyopus fehlmanni*					*Smilosicyopus chloe*				
*Region*	*Archipelago*	n	*N*	*H_d_*	π	theta S	n	*N*	*H_d_*	π	theta S	n	*N*	*H_d_*	π	theta S
Southwest	*Vanuatu*	14	8	0.769	0.0025	3.476	3	2	–	–	–	11	8	0.945	0.0039	4.142
Pacific	*New Caledonia*	52	34	0.906	0.0044	11.002	5	5	1	0.0033	2.404	33	22	0.962	0.0047	7.332
	*Fiji*	2	2	–	–	–	–	–	–	–	–	–	–	–	–	–
	*Australia*	–	–	–	–	–	2	2	–	–	–	–	–	–	–	–
	*Subtotal*	68	40	0.885	0.0039	10.857	10	9	0.900	0.0036	3.315	–	–	–	–	–
Northwest	*Papua*	36	24	0.908	0.0098	7.219	5	3	0.7	0.0021	1.438	–	–	–	–	–
Pacific	*Palau*	9	7	0.917	0.0070	3.679	6	5	0.933	0.0049	3.523	–	–	–	–	–
	*Philippines*	22	13	0.892	0.0155	5.494	–	–	–	–	–	–	–	–	–	–
	*Japan*	29	15	0.771	0.0063	8.043	–	–	–	–	–	–	–	–	–	–
	*Subtotal*	96	43	0.865	0.0098	9.324	11	8	0.978	0.0031	2.048	–	–	–	–	–
Total		164	75	0.937	0.0129	15.519	21	14	0.900	0.0036	3.929	44	26	0.96	0.046	7.805

Sampling locations and number of individuals sequenced (n) are listed. Number of haplotypes (*N*), haplotype diversity (*H_d_*), nucleotide diversity (π) and mean number of segregating sites between sequences (S) are listed for population presenting at least 5 individuals. Instances of no data are indicated (–).

### Phylogeographic Analyses

#### Sicyopus zosterophorum

**Figure 2:**
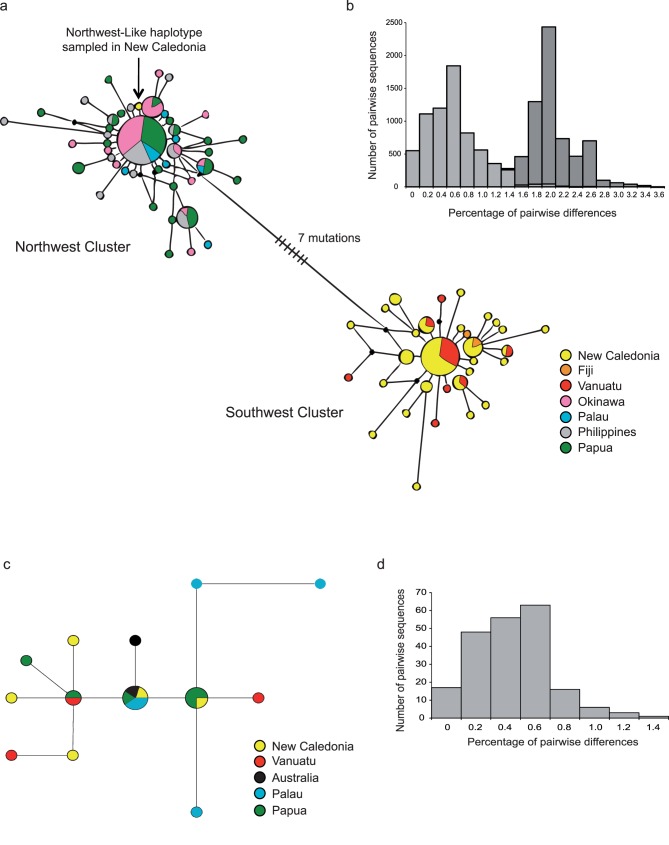
Median-joining networks and mismatch distributions for *Sicyopus zosterophorum* and *Smilosicyopus fehlmanni*. (a) Median-joining network of *Si. zosterophorum* constructed using the program Network 4.1 [57] using 670 pb of COI from 164 sequences; (b) Mismatch distribution of *Si. zosterophorum* overall COI genetic sequences; (c) Median-joining network of *Sm. fehlmanni* constructed using the program Network 4.1 [57] using 670 pb of COI from 21 sequences; (d) Mismatch distribution of *Sm. fehlmanni* overall COI genetic sequences.

There was clear evidence of a genetic break within the widespread distribution of this species in the Central West Pacific. From COI, the distribution of pairwise genetic distances was bimodal and exhibited two peaks at around 0.6 and 2.0% of pairwise differences (Fig. 2b). The first mode comprised pairwise genetic distances between specimens collected either in the northwest or in the southwest Pacific. The second mode included pairwise genetic distances between specimens from the northwest Pacific compared to specimens from the southwest Pacific.

Network analysis confirmed the presence of two genetic groups. The COI median-joining network (MJN) revealed two genetic clusters of respectively 31 and 43 haplotypes (Fig. 2a). These two genetic groups were separated by a minimum of seven nucleotide substitutions (Fig. 2a). The first group was restricted to the northwest region including Japan, Palau, Philippine and Papua and the second to the southwest region including New Caledonia, Vanuatu and Fiji. However, one northwest haplotype, represented by one individual, occurred within the southwest Pacific group (Fig. 2a). This result was double-checked and we believe that it was not due to an error in the field or in the laboratory. Within these two regions, each cluster exhibited a star-like topology with no obvious geographic pattern. The major haplotype in the northwest cluster was shared by individuals from four localities (Japan, Palau, Philippines and Papua). The major haplotype in the southwest cluster was shared by individuals from Vanuatu and New Caledonia but not individuals from Fiji. The genetic structure revealed by the COI network was consistent with a Cytb network (Cytb data and network not shown).

The AMOVA performed among the six populations (Japan, Philippines, Palau, Papua, New Caledonia and Vanuatu) showed highly significant genetic differentiation (φ_ST_ = 0.55737, p-value <10^−5^, Table 3). This differentiation was driven by the comparison between the northwest and southwest clusters; as among populations φ_ST_ within each cluster showed no significant values (Table 3). To increase statistical power of analysis, the AMOVA was then performed on a grouped dataset. We pooled individuals from the northwest cluster and individuals from the southwest cluster. Thus, on the basis of geographic proximity, two groups of specimens were considered: (1) Japan, Philippines, Palau and Papua (2) New Caledonia, Vanuatu and Fiji. The AMOVA between these two groups showed a significant genetic differentiation (φ_ST_ = 0.6743, p-value <10^−5^, Table 4). Pairwise φ_ST_ revealed a contrast between intra-group and inter-group genetic differentiation. Both geographic groups (*i.e.* the northwest and the southwest), exhibited no significant genetic differentiation (0.16216<pairwise φ_ST_ p-values <0.9009). On the contrary, pairwise φ_ST_ values between the two groups were highly significant (p-value <10^−5^) (Table 5).

**Table 3:** Hierarchical partitioning of molecular variation within and among all populations (AMOVA) based on COI haplotypes for *Sicyopus zosterophorum* and *Smilosicyopus fehlmanni*.

	Source of variation	d.l.	Variance component	φ_ST_	p-value
*Sm. fehlmanni*	Among populations	4	0.21235	0.21830	0.01955±0.00517
	Within populations	16	0.76042		
*Si. zosterophorum*	Among populations	6	3.14897	0.55737	<10^−5^
	Within populations	158	2.50073		

**Table 4:** Hierarchical partitioning of molecular variation (AMOVA) based on COI haplotypes within and among southwest and northwest Pacific groups and populations for *Si. zosterophorum*.

	Source of variation	d.l.	Variance component	φ_ST_	p-value
*Si. zosterophorum*	Among groups	1	5.15123	0.67502	0.02835
	Among population within groups	5	−0.01383	−0.00558	0.59433
	Within populations	158	2.49382	0.67321	<10^−5^

**Table 5:** Pairwise F-statistics for 6 populations of *Sicyopus zosterophorum*.

	Vanuatu	NC	Japan	Papua	Philippines	Palau
Vanuatu	−	0.666±0.051	0.000±0.00	0.000±0.00	0.000±0.00	0.000±0.00
NC	−0.008	−	0.000±0.00	0.000±0.00	0.000±0.00	0.000±0.00
Japan	0.770	0.760	−	0.162±0.029	0.468±0.048	0.423±0.052
Papua	0.661	0.685	0.023	−	0.558±0.061	0.966±0.014
Philippines	0.606	0.668	−0.003	−0.009	−	0.900±0.023
Palau	0.781	0.748	−0.007	−0.050	−0.045	−

Pairwise φ_ST_ for COI data are below diagonal and p-values are above diagonal. Only populations with more than 5 individuals were taken into account. Populations are labeled according to Table 1 (country).

#### Smilosicyopus fehlmanni

In pronounced contrast to the co-distributed and biologically similar *Si. zosterophorum*, there was no evidence of a genetic break in this species. The distribution of pairwise COI genetic distances was unimodal (Fig. 2d). The MJN showed no evidence of genetic structure among the species range and specimens from distant locations shared the same haplotypes (Fig. 2c). Individuals sampled in different locations shared the most frequent haplotype. The lack of genetic structure revealed from the COI network was reflected by a lack of structure in the Cytb network (data not shown).

The AMOVA performed among three populations (Palau, Papua and New Caledonia) showed no genetic differentiation (φ_ST_ = 0.0251, p-value = 0.3), Table 3). This result is supported by the non-significance of pairwise φ_ST_ calculated between the populations (New Caledonia, Papua and Palau) (Table 6).

**Table 6:** Pairwise F-statistics for 3 populations of *Smilosicyopus fehlmanni*.

	New Caledonia	Papua	Palau
New Caledonia	−	0.360±0.066	0.252±0.042
Papua	0.062	−	0.738±0.040
Palau	0.069	−0.058	−

Pairwise φ_ST_ for COI data are below diagonal and p-values are above diagonal. Only populations with more than 5 individuals were taken into account. Populations are labeled according to Table 1 (country).

### Species Identity

**Figure 3:**
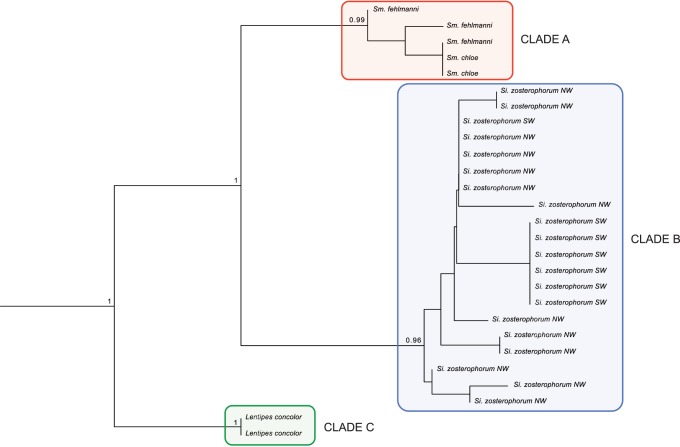
Phylogenetic relationship between *Sicyopus zosterophorum*, *Smilosicyopus fehlmanni* and *Smilosicyopus chloe*. The rooted phylogram was drawn using 760-joining methods using Kimura two-parameter distances (K2P). NW and SW refers to northwest and southwest biogeographic regions within *Si. Zosterophorum* samples.

The phylogram constructed using the Rh gene allowed us to test whether the two mitochondrial groups of *Si. zosterophorum* were cryptic species. The data set consisted of 27 sequences of the nuclear Rh gene that were obtained from individuals of the two mitochondrial groups of *Si. zosterophorum* (13 for the northwest group and six for the southwest group) plus three individuals of *Sm. fehlmanni*, two of *Sm. chloe* and two of the outgroup *Lentipes concolor*. The phylogram showed three clades (A, B and C), which were highly supported by bootstrap values. Clade A included the two congeneric species (*Sm. fehlmanni* and *Sm. chloe*). Clade B included all specimens of *Si. zosterophorum* (Fig. 3). Finally, clade C consisted of the outgroup *Lentipes concolor*. Within clade B, the samples of *Si. zosterophorum* did not constitute reciprocally monophyletic geographic clades (Fig. 3).

### Demography

**Figure 4:**
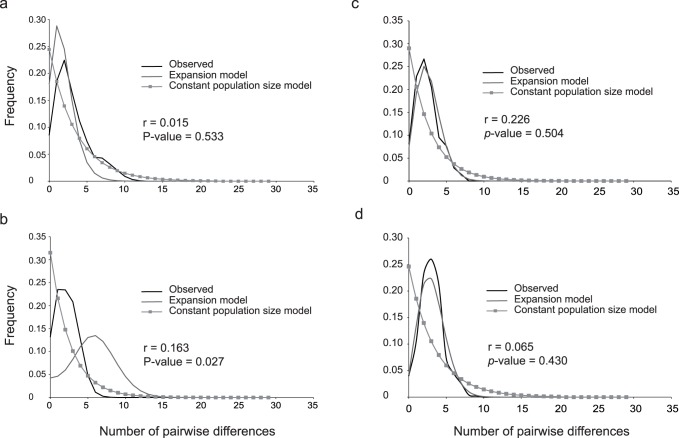
Mismatch distribution for observed data compared with data expected under a demographic expansion and a constant population size model. (a) *Sicyopus zosterophorum* southwest clade; (b) *Sicyopus zosterophorum* northwest clade; (c) *Smilosicyopus fehlmanni*; (d) *Smilosicyopus chloe*. Using the raggedness test, population expansion was rejected for the northwest samples of *Si. zosterophorum* (P = 0.027) but could not be rejected for the southwest samples of *Si. zosterophorum* (P = 0.533), *Sm. Fehlmanni* (P = 0.504) and *Sm. Chloe* (P = 0.430) samples.

Three types of analyses were employed to gain insight into the demographic history of populations of the three species. For the northwest cluster (Fig. 4b) of *Si. zosterophorum*, the observed raggedness value of 0.163 was slightly significantly smoother (p-value = 0.027) than mismatch distributions simulated from the expansion population size model. This was not the case for the southwest cluster (Fig. 4a; *r* = 0.015, p-value = 0.533), which means that the hypothesis of expansion cannot be rejected. Fu’s *Fs* value was strongly and slightly significantly negative for the southwest cluster (*Fs* = −14.972, p-value = 0.00424) whereas no significant Fu’s *Fs* was found for the northwest cluster (*Fs* = −2.828, p-value = 0.2197) (Table 7).

**Table 7:** Demographic parameters for the species *Sicyopus zosterophorum*, *Smilosicyopus fehlmanni* and *Smilosicyopus chloe*.

	Fu’s *Fs*	*r*	τ	θ_0_	θ_1_
*Sm. chloe*	−9.69 (0.00450)	0.065 (0.430)	3.15	0.00	99,999
*Sm. fehlmani*	∞	0.226 (0.504)	2.60	0.00	40,005
*Si. zosterophorum*					
Northwest	−2.82 (0.21975)	0.163 (0.027)	6.56	0.00	23.07
Southwest	−14.97 (0.00424)	0.015 (0.533)	1.19	0.68	33,334

Fu’s *Fs* value and corresponding p-value, Harpending’s raggedness indices (*r*) with corresponding p-value and mismatch parameters τ, θ_0_ and θ_1_ as reported in Arlequin [63] are listed. Values that could not be resolved are designed by infinity sign (∞).

We also used coalescence time to predict the time when the two clades of *Si. zosterophorum* diverged. Parameter estimates for a coalescent model of constant population size were equal or very similar for all three replicates. All effective sample size values were greater than 200 and plots of parameter trends indicated sufficient mixing among chains. The heuristic estimate for *t*, the time of population splitting, was 306,000 years (306 Kyr) ago (95% HPD 79–625 Kyr BP).

For *Sm. fehlmanni*, the observed raggedness value of 0.226 was not significantly smoother than mismatch distributions simulated from expansion model populations (p-value = 0.504) (Fig. 4c, Table 7), which means that the hypothesis of expansion cannot be rejected.

For *Sm. chloe*, the observed raggedness value of 0.065 was not significantly smoother than mismatch distributions simulated from expansion model populations (p-value = 0.430) (Fig. 4d, Table 6). Fu’s *Fs* value was strongly and significantly negative (*Fs* = −9.69337; p-value = 0.0045) (Table 7). The two demographic parameters suggest that the population might be undergoing expansion.

## Discussion

### Contrasting Patterns of Genetic Structure

Because of their extended PLD (about 55 days) populations of neither goby species (*Si. zosterophorum* and *Sm. fehlmanni*) were expected to show genetic breaks within the Central West Pacific bioregion. Surprisingly, the analysis of population genetics over the sampled region revealed contrasting patterns of genetic structure between the two species studied. Despite low number of samples, genetic connectivity among *Sm. fehlmanni* populations was high as expected, suggesting that for this species, neither the fragmentation of freshwater habitat nor the oceanic currents surrounding the Central West Pacific Ocean or the presence of the Torres Strait barrier have created significant barriers to gene exchange. Likewise, the broadly distributed amphidromous goby *Sicyopterus lagocephalus*, displays low genetic structure among the islands of the West Pacific [24]. These results also are consistent with patterns of high connectivity observed in amphidromous species such as Neritidae and Neritiliidae snails [27], [74], [75], [76], Galaxiidae [77] or Sicydiinae fish [24], [78], [79]. However, a larger number of samples for *Sm. fehlmanni* will be necessary to confirm these results in future studies.

By contrast, the haplotype networks of *Si. zosterophorum* displayed a deep division into two haplogroups. One group was restricted to the northwest of the Central West Pacific, and the other group was restricted to the southwest. The phylogenetic tree (Fig. 2), based on the nuclear Rh gene, showed that intraspecific divergence of *Si. zosterophorum* individuals was similar to interspecific divergence between *Sm. fehlmanni* and *Sm. chloe*, suggesting that the two haplogroups may be cryptic species. But, there are three arguments against this. The first is that *Si. zosterophorum* individuals from northwest and southwest were not completely reciprocally monophyletic as one northwest Pacific-like haplotype was sampled amongst 68 samples from the southwest Pacific. Secondly, there were no morphological or meristic differences between the individuals of the two bioregions. And lastly, divergence among the putative cryptic species was not reflected in the rhodopsin phylogeny, although the lack of rhodopsin geographic clades could be due to retained ancestral polymorphism. Considering these arguments, we treated all specimens of *Si. zosterophorum* as belonging to a single species.

The genetic distance between the two groups indicates the presence of one or more barriers to dispersal that may have prevented, or be preventing, migration between these geographic areas. However, the occurrence of one northwest Pacific-like haplotype in one of the 68 southwest Pacific samples suggests that migration and gene flow may have been possible (now or at some time in the evolutionary past) between the two geographic areas, but not sufficient to ensure mtDNA homogenization among regions. A similar pattern has been found for the diadromous species *Galaxias maculatus* between Tasmania and New Zealand suggesting a directional rare dispersal event [80].

### Putative Effect of the Torres Strait Barrier

The location of the genetic break in *Si. zosterophorum* appears to correspond with the Torres Strait barrier (TSB). This narrow passage connects the northerly and southerly parts of the distribution of *Si. zosterophorum* and *Sm. fehlmanni* in the Central West Pacific. The species are not known to occur in streams on the northern coastline of Papua New Guinea [44] where the specific requirements for their establishment (short, steep, well oxygenated streams) are known to be scarce or absent in this region. Also, the oceanic currents along the northern coastline of Papua New Guinea do not favour the distribution of larvae in this region. The allopatric nature of *Si. zosterophorum* clades across the TSB has been documented in other clades of taxa such as reef fish and other marine organisms like crustaceans, molluscs, echinoderms and sharks [25], [30], [37], [81], [82] even though some of these taxa could have dispersed to the north of Papua land mass. In the tropical Indo-Pacific province, it has been suggested that during the Pleistocene, lowering of sea levels repeatedly changed the extent of the TSB [5], [37], [81]. The estimated time of divergence between the northwest and southwest clades of *Si. zosterophorum* (*i.e.* 306 Kyr; 95% HPD [79; 625]) is concordant with the most recent closure of the intermittent TSB during the Pleistocene, which is further evidence that the TSB is implicated in the genetic break in this species. Earlier closures of Torres Strait are known, which may have also led to divergence between the clades. Sea level has been at least 10 m below present level during 91% of the time for the last 250,000 years [7] (Fig. 1b) and during this time Papua New Guinea would have been largely connected to the Australian mainland at the Torres Strait [83]. This scenario of vicariant events across the TSB with the persistence of sister populations on either side of the land bridge is likely for *Si. zosterophorum*, although other factors such as life history traits, oceanic barriers or habitat availability may have played important roles and cannot be ruled out.

It appears that similar processes have not resulted in genetic divergence in *Sm. fehlmanni*. Despite sea level fluctuations, *Sm. fehlmanni* shows no phylogeographical structure and numerous haplotypes are shared between geographically distant populations, which indicates high connectivity. One hypothesis for the lack of phylogeographical structure across the TSB for *Sm. fehlmanni* is that one of the two putative lineages formed by a TSB vicariant event may have been lost due to local extinction on one side of the Torres Strait, followed by recent re-colonization through the Torres Strait within the last 7,000 years [82]. In this case, we would expect to find no appreciable genetic divergence between populations and reduced diversity in the colonized region relative to the source [84]. This is indistinguishable from a second hypothesis where the range of this species did not span the TSB, but has subsequently expanded as a consequence of Pleistocene sea level fluctuations. A final hypothesis is that populations on both sides of the Torres Strait may have been linked *via* northern Papua New Guinea even when the TSB was closed. However, the coastal upwelling along the north coast of Papua New Guinea induces cooling of the sea surface temperatures associated with the Pacific warm pool (5°S–5°N, 140°E–150°E) [85], which may not favour larval dispersal through this area. It is also possible that *Sm. fehlmanni* exhibits weak differentiation across the TSB, but our small sample sizes limited our ability to detect genetic structure. Additional analyses of this species are needed to test these alternative hypotheses.

### Demography and Hypotheses

The demographic patterns inferred here provide greater understanding of the evolution and biogeography of the Sicydiinae. Clades of *Si. zosterophorum* revealed contrasting patterns. The southwest group had strongly negative and significant *Fs* values indicating non-equilibrium population dynamics. There was evidence of an excess of low frequency haplotypes as expected from a recent population expansion or secondary contact between previously allopatric populations [86]. By contrast, no statistical evidence of expansion in population size was found for the northwest group. However, the statistical support for expansion of the southwest cluster is quite weak (*Fs* values only slightly significant) and the sampling bias between the two clusters (more individuals in the northwest than in the southwest as well as unequal population size within southwest cluster) affects the robustness of these statistical analyses. Additional data, including a larger number of individuals and the use of additional polymorphic nuclear markers, are required to strengthen our interpretations.

The signature of dynamic expansion among populations of the two congenerics *Sm. chloe* and *Sm. fehlmanni* cannot be rejected. These two species co-occur in the rivers of the Vanuatu and New Caledonia islands, showing similar habitat preferences (*i.e.* from the middle to the upper courses of rivers and prefer swift, clear and high gradient streams with rocky and boulder strewn bottoms [43], [48]); yet *Sm. chloe* presents a geographically restricted species range, while *Sm. fehlmanni* is much more widespread. One possible explanation for their different range size would be the ‘taxon cycle hypothesis’ [87], which suggests that over ecological and evolutionary time scales, species progress through predictable sequential periods of range expansion and contraction that are accompanied by associated ecological shifts. This hypothesis has been postulated to characterize dynamic biogeographic histories in various island communities including lizards [88], birds [89], ants [90] and amphidromous species [91]. Here, the two species of *Smilosicyopus* studied might be at different taxon cycle stages (*i.e.* different population dynamic in relation with their particular distribution range). *Sm. chloe* would be in an early stage (with a restricted distribution, [87]) and experiencing a secondary expansion phase (see the negative and significant Fu’s *Fs*) and *Sm. fehlmanni* would be in an older stage (with a widespread distribution, [87]). From a niche-based perspective [92] and given their co-occurrences, it is possible that *Sm*. *fehlmanni* competitively excluded *Sm. chloe* toward a marginal, geographically restricted, ecological niche.

Demographic analyses for the southwest cluster of *Si. zosterophorum* as well as for *Sm. chloe* and *Sm. fehlmanni* populations, provide some evidence that these populations are in an expansion phase. If this is the case, two non-exclusive hypotheses could be raised, which could be tested in further studies. First, populations may be expanding following a past bottleneck caused by population depletion, a selective event, or a founder event associated with colonization of isolated islands [27], [65], [93], [94]. Such patterns are commonly found in highly dispersive organisms inhabiting spatially fragmented or ephemeral habitats. This has been observed in several widespread reef fish species, including for example *Lutjanus fulvus* [5], *Naso unicornis* [34], *Scarus psittacus* [95], and in amphidromous species living on oceanic islands [24], [74], [96]. Secondly, there could have been wholesale migration from one bioregion to another. Exploitation of vacant habitat in the recently colonized bioregion may lead to a signature of expansion. Genetic divergence between populations of *Si. zosterophorum* was too great to use statistical models to test this hypothesis on the current dataset.

## Concluding Remarks

In the Central West Pacific, we have found that two widespread amphidromous Sicydiinae species with similar PLD and biology display contrasting patterns of genetic structure. Our data, in addition to similar studies in this region, suggests that a Pleistocene barrier to marine dispersal (the Torres Strait) may have shaped the genetic pattern of one of the species (*Si. zosterophorum*). Other factors, however, must be taken into account else both species would exhibit similar levels of genetic differentiation across the Torres Strait. These may include subtle differences in dispersal and colonization ability, and habitat requirements, which may have direct or indirect effects on genetic connectivity now and in the past. The rarity and widespread nature of the studied species on remote islands makes further research in these areas a difficult task. Additional genetic data for the two widespread species from other regions of their range, especially closer to either side of the Torres Strait (*e.g.*, in the Solomon Islands and south-eastern Indonesia), would nonetheless help clarify the extent to which Pleistocene events have influenced the phylogeography of amphidromous species. Our finding suggest that amphidromous species that are widely distributed may harbor cryptic evolutionary lineages. Indeed, the two widespread gobies with similar distributions have differing levels of population structure, implying that one type of conservation action will not fit all. Because Sicydiinae species are rare and susceptible to anthropogenic and environmental perturbations, strategies aiming to conserve them should consider the presence of possible cryptic evolutionary lineages and cannot rely on a widespread population distribution with high-supposed dispersal abilities to prevent stock depletion.
